# Supplementary material for: The genome sequence of the Beautiful Gothic, Leucochlaena oditis (Hübner, 1822) (Lepidoptera: Noctuidae)
Source: Wellcome Open Res. 2026 Sep 14;11:661. [Version 1] doi: 10.12688/wellcomeopenres.27816.1 (PMC13768708; doi:10.12688/wellcomeopenres.27816.1)
Supplement: Peer review history

# Reviewer report: Reviewer response for version 1

**DOI:** 10.21956/wellcomeopenres.30609.r175151

**Published:** 2026-11-13

**Authors:** Wai Lok So

**Affiliations:** 1 The Hang Seng University of Hong Kong, Hong Kong, Hong Kong

The manuscript presents a high-quality genome assembly of *Leucochlaena oditis* as part of the Project Psyche initiative. The assembly methods are technically sound with excellent scaffolding of haplotype 1, including both W and Z chromosomes and a complete mitochondrial genome.

 The data are publicly available and transparent, with Accession number PRJEB82296.

 The three-part EBP metric, calculated for the haplotype 1 assembly, is 6.C.Q67, which passes the 6.C.Q40 reference standard.

 The combined haplotype assemblies have a 97.29% of k-mer completeness. The BUSCO value passes by having 98.9% for haplotype 1 and 92.7% for haplotype 2.

Are the datasets clearly presented in a usable and accessible format, and the assembly and annotation available in an appropriate subject-specific repository?

Yes

Are sufficient details of the sequencing and extraction, software used, and materials provided to allow replication by others?

Yes

Are the rationale for sequencing the genome and the species significance clearly described?

Yes

Are the protocols appropriate and is the work technically sound?

Yes

Reviewer Expertise:

Evolution, Genomics, Arthropods, Myriapods

I confirm that I have read this submission and believe that I have an appropriate level of expertise to confirm that it is of an acceptable scientific standard.

# Reviewer report: Reviewer response for version 1

**Published:** 2026-09-14

**Authors:** Automated Benchmarking

**Affiliations:** 1 Wellcome Sanger Institute, Hinxton, UK

As this article is a Genome Note, Automated Benchmarking has been used to determine whether the reported assembly passes three defined checks established by the Earth BioGenome Project: the raw and assembled data are publicly available, the Earth BioGenome Project three-part metric is met, and the BUSCO score passes the stated threshold. For Leucochlaena oditis (ilLeuOdit1), all three checks passed. Data available: pass. ENA study PRJEB82296, raw-read accessions ERR13946509 and ERR13947537, and assembly accessions GCA_964656205.1 and GCA_964656185.1 are public. Earth BioGenome Project three-part metric: pass. Haplotype 1 is reported as 6.C.Q67, meeting the 6.C.Q40 reference standard. BUSCO: pass. Haplotype 1, which is proposed as the reference assembly for this species, has 98.9% complete BUSCOs (98.4% single-copy and 0.5% duplicated). BUSCO software version: 5.5.0; lineage dataset: lepidoptera_odb10. It meets the target of greater than 90% complete and single-copy BUSCOs. This decision uses values reported in the Genome Note and public accession records.

Reviewer Expertise:

NA

I confirm that I have read this submission and believe that I have an appropriate level of expertise to confirm that it is of an acceptable scientific standard.
